# Laboratory Study of the Effect of Zeolite and Cement Compound on the Unconfined Compressive Strength of a Stabilized Base Layer of Road Pavement

**DOI:** 10.3390/ma15227981

**Published:** 2022-11-11

**Authors:** Amin Sheikh, Mahdi Akbari, Gholamali Shafabakhsh

**Affiliations:** Faculty of Civil Engineering, Semnan University, Semnan 35131-19111, Iran

**Keywords:** soil stabilization, environmental protection, cement, zeolite, compressive strength, SEM-EDX, XRD, failure strain

## Abstract

Soil stabilization using cement is regarded as one of the conventional methods to improve the engineering properties of soil used in infrastructure and road bodies. Considering the environmental problems caused by the production and consumption of cement, finding a suitable replacement for cement is necessary. The present study aims to experimentally evaluate the effect of using zeolite instead of cement in the stabilization of pavement layers. In this research, only 5% of cement was used in the control sample, while zeolite was used instead of cement in other samples by 20, 30, 40, and 60 wt.% of cement. According to the analysis, the highest unconfined compressive strength was obtained in the sample containing 30% (wt.% of cement) of zeolite instead of cement (equivalent to 1.5% of the total stabilizing materials) after 28 days of treatment, which was 29% more than that of the sample without zeolite. Evaluating the fracture strains reveals that using zeolite instead of cement increases the fracture strain by 33%, and in other words, changes the behavior of the sample from brittle mode to soft mode.

## 1. Introduction

The development of transportation and the expansion of road networks (including freeways and highways) are among the most important indicators of development in a country. Many experts believe that the expansion of road networks can affect the growth and expansion of other development factors and provides the basis for development in different directions. However, the presence of weak soils in the road infrastructure is one of the great challenges in this regard. On the other hand, a road with a more resistant bed allows for a decrease in the thickness of the pavement layer and reduces costs [1,2].

The existence of weak infrastructure layers, or the lack of suitable borrowed materials for constructing pavement layers, is one of the reasons for rapid pavement deterioration. The occurrence of settlements or permanent deformations in pavement layers indicates road deterioration, which overshadows traffic safety and increases user costs [3]. So far, various methods have been introduced to improve and enhance the performance of the underlying, body, and pavement layers, which include stabilization, consolidation, reinforcement, or even replacement of materials [4,5,6,7,8]. The use of waste materials of aluminosilicate origin can be one of the suitable solutions for stabilizing applications. However, on the other hand, the abundance of natural materials with pozzolanic properties in the nature of the central region of Iran has brought the opportunity to reduce as much as possible the consumption of materials such as cement, which is an expensive production process and with high energy consumption. Most road construction industry researchers use pozzolanic materials such as cement to stabilize subgrade soil or the pavement body (including base and subbase layers). Generally, different percentages of cement (usually between 2 and 10%) are used to stabilize the soil with cement, considering the grain size, soil type, and the intended purpose of stabilization. For example, an amount of cement of less than 5% of the dry soil weight does not have a significant effect on improving the soil characteristics, while amounts of more than 5% are not economical from an environmental or economic point of view, because of the high volume of cement used in road construction projects. Furthermore, operational experience states that an amount of 5% cement is more common, and this ratio of cement weight to dry soil weight is used to stabilize the soil utilized in the infrastructure or pavement layers in most projects.

In recent decades, a large body of research has been conducted to find alternative pozzolanic materials in soil stabilization, due to the environmental problems related to the production and consumption of cement. Further, cement-stabilized layers have brittle behavior and are sensitive to overload [9,10,11,12].

Zeolite has pozzolanic properties and is mainly composed of aluminosilicate. This mineral is used as a natural pozzolan to protect the environment [13,14,15,16]. So far, various studies have been carried out to investigate the effect of replacing a part of cement with zeolite in soil stabilization [12,13,17,18].

Mola-Abasi and Shooshpasha [17] evaluated the effect of using different percentages of zeolite to replace cement in sands stabilized with cement in a laboratory. In this research, soil stabilizers included type 2 cement with percentages of 2, 4, 6, and 8 and zeolite with replacement percentages of 0, 10, 30, 50, 70, and 90. The unconfined compressive strength of the samples was measured at different relative densities, including 50, 70, and 85%, in 7- and 28-day treatment periods. The results showed that replacing 30% zeolite instead of cement decreased the unconfined compressive strength of the samples after 7 days of treatment, although it increased between 20 and 80% for different percentages of zeolite in both 28 and 90 days. Moreover, the efficiency of using zeolite increases by increasing soil porosity [17]. The results also show that the mixture of soil and cement has a brittle behavior, and the addition of zeolite increases the amount of strain and leads to a softer behavior in the soil. The sample containing 8% cement, which has 50% zeolite and has been treated for 28 days, tolerates stress of 130 kPa with a strain of 3.3 mm. However, the maximum resistance in samples without zeolite is 90 kPa, breaking at a strain of 3 mm. Research in this regard was conducted only on Babolsar sand, which is called poorly grained sand, for whom 100% of its grains are smaller than one millimeter, according to the classification of the unified method [17,19].

Mariri, Ziaie Moayed, and Kordnaeij [18] studied the effect of using zeolite instead of cement, along with recycled polyester fibers (PET), on the unconfined compressive strength of collapsing soils in a laboratory. Based on the results, the optimal amount of zeolite to replace cement is equal to 10 and 30% in samples containing 4 and 8% of cement, respectively. Further, the addition of fibers increases the fracture strain and causes samples to show soft behavior rather than brittle behavior. This research shows that adding water to samples by 20% more than the optimum moisture percentage could increase the unconfined compressive strength, indicating more water absorption in the samples containing zeolite. The soil used in this research is collapsing clay soil, prepared from Kalaleh city in the Golestan province of Iran, and is classified as CL according to unified classification [18].

Shi [20] investigated the effect of a mixture of cement and zeolite in stabilizing two types of sandy and silty clay soil by performing an unconfined compression test. In this study, the percentages of cement and zeolite were considered 2.5, 5, and 10%, and the samples were treated for 7, 28, and 90 days. Meanwhile, a ratio of 9 to 1 was selected in the combination of cement and zeolite. The results highlighted that the highest compressive strength obtained in the samples with silty clay and sandy soil was equal to 1.28 and 7.65 MPa, respectively, which was achieved with 10% additive and during 90 days of treatment. The value of 1.28 MPa for unconfined compressive strength exceeds the minimum value reported in the current US Code (350 kPa). By using an equal percentage in the combination of zeolite and cement and considering the same treatment time for both combinations, the mixture of zeolite and cement has a greater effect on increasing soil strength compared to the combination of powdered ash and cement [20].

Wu et al. [8] studied the engineering properties of using modified synthetic zeolite additive in cement-stabilized sand materials and evaluated the effect of different ratios of cement content and modified synthetic zeolite as additives on two important parameters of pavement design, i.e., stiffness and fatigue. The greater stiffness of the base layer stabilized with cement causes a wider distribution of the loads, and therefore, the stresses decrease significantly at lower depths. On the other hand, greater fatigue resistance prevents the creation and growth of cracks caused by accumulated traffic loads. In this research, a four-point bending test was conducted to assess the performance of the cement-stabilized base layer under repeated compression and tension. The results indicated that adding a certain amount of zeolite to the cement-stabilized sand mixture increases the sample’s resistance to fracture. Fatigue relations for all tested mixtures were obtained by plotting load cycles up to the failure time as a function of applied stress or initial strain levels [8].

Ahmadi Chenarboni et al. [21] evaluated the effect of the relative replacement of cement with zeolite on the mechanical behavior of the soil, aiming to reduce the damages caused by the change in the volume of clay soils under different moistures. Researchers selected four different percentages of cement (6, 8, 10, and 12%) and different percentages of zeolite to replace cement (0, 10, 30, 50, 70, and 90%). Then, a standard compression test was conducted. According to the results, adding cement leads to an increase in maximum dry density (MDD) and optimum moisture content (OMC) of the soil and cement mixture., although increasing the ratio of zeolite leads to opposite trends [21].

ShahriarKian et al. [22] studied the performance of silty sand soil stabilized with a combination of cement and zeolite in wet and drying cycles using an unconfined compressive strength test in freeze and thaw cycles. Based on the results, zeolite along with cement can be used as a stabilizer to improve the mechanical behavior of soil against freeze and thaw cycles. Moreover, an increase in the number of freeze and thaw cycles decreases the strength of the sample in general, while the addition of 3 to 9% zeolite increases the durability of the sample against the freeze and thaw cycles. Finally, the sample containing 6% cement and 9% zeolite has the highest resistance to freeze and thaw cycles by a small difference.

The environmental problems caused by the production and consumption of cement are one of the most important reasons for using zeolite instead of cement. Regarding the subject of this study and considering the low cost and easy accessibility of zeolite in the central region of Iran, it could be stated that there are many reasons for using zeolite to replace cement, some of the most important of which are as follows:

- Reducing construction costs by reducing cement consumption.
- Reducing fuel consumption by reducing cement consumption.
- Reducing the emission of pollution by reducing the consumption of cement.
- Shortening the process of supplying materials, due to the use of zeolite raw materials with minimal production processes (unlike cement production, which requires considerable time for the process of supplying raw materials to the production of the final product).
- Abundance and easy availability of zeolite mineral in the nature of the central part of Iran.
- The lower price of zeolite than cement.

In the literature, only problematic, weak, or small-size soil aggregates were investigated, which were implemented as subgrade soils. In fact, they were focused on using zeolite as a suitable material to improve the subgrade layer of roads, not the pavement body layers. In this research, the maximum grain size is equal to 19 mm. The aggregate with a maximum grain size of 19 mm is one of the conventional aggregate materials used in the base layer of pavements. As aggregate materials for pavement layers, the amount of small-size aggregates (especially clay and silt) was limited due to the probability of structural failure when exposed to water [23].

Despite the relatively acceptable resistance of this type of soil, if it is improved (as a base layer), some advantages could be provided: the total thickness of the pavement could be significantly reduced (especially under heavy traffic loads), higher tensions due to traffic loads could be applied, the durability of the implemented base layer could be extended, and so on.

The main objectives of the present study can be stated as follows: determining the effectiveness of using zeolite powder (aggregates passed sieve No. 200) as an alternative to cement, in stabilizing soil layers of road pavement; determining the optimal percentage of zeolite to replace cement (assuming a constant weight of stabilizing materials of about 5% of the weight of soil materials); investigating the state of resistance and strain changes of soil mixtures stabilized with zeolite and cement compounds.

Despite conducting various studies to investigate the effect of using zeolite instead of cement in the stabilization process of problematic or fine-grained soils, it is still necessary to study the stabilization of consumables in pavement layers using zeolite. In past research, soils with a maximum grain size of 1 mm were usually investigated. This research seeks to evaluate the effectiveness of replacing a part of cement with zeolite in the stabilization of pavement base layers with a maximum grain size of 19 mm, which is one of the conventional soils used in the base layer. The purpose of this research is to determine the optimal percentage of replacement and the proposed relationship to stabilize pavement layers with zeolite, as well as to specify the behavior of the stabilized soil, by analyzing the results of compressive strength tests, scanning electron microscopy (SEM-EDX), and X-ray diffraction analysis (XRD) images.

## 2. Materials and Methods

Typical layers of a conventional flexible pavement include seal coat, surface course, tack coat, binder course, prime coat, base course, subbase course, compacted subgrade, and natural subgrade. The first 5 elements include 3 bituminous coating layers and 2 layers of asphalt mixture. However, the other 4 elements are mostly composed of aggregate materials. In some road construction projects, due to various technical reasons, it is necessary to stabilize the base and subbase layers (or even subgrades) using physical, mechanical, chemical, or mineralogical methods.

In previous studies, various types of research have been conducted on the use of zeolite as a modifier and replacement for filler or aggregate materials in asphalt mixtures. In addition to asphalt mixtures, studies have also been conducted on the applications of zeolite to stabilize subgrade layers (especially subgrades with loose and soft soils).

What is relevant about the base and subbase layers is that the subbase layer is mainly implemented in pavements with very high traffic loads or with a soft subgrade, but the base layer is among the main layers that have to be implemented in the hot mix asphalt concrete pavements. Therefore, according to the existing limitations, in the present study, only one type of aggregate material from the base layer has been studied, and its specifications are provided in the relevant section of the text.

**Figure 1:**
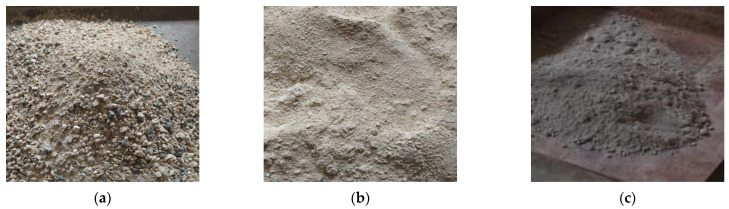
Consumable materials of the research include (**a**) aggregate materials of the base layer, (**b**) zeolite, and (**c**) cement.

The materials used in this research include soil used in pavement layers, cement, and zeolite as an additive. Figure 1 illustrates a schematic view of soil, zeolite, and cement used in this research.

### 2.1. Aggregate Materials for Pavement Layers

**Figure 2:**
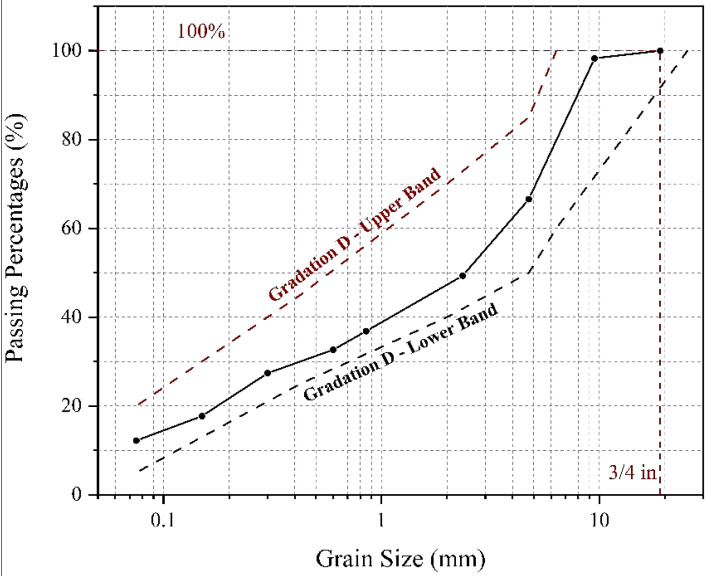
Granulation diagram of the studied soil.

The soil studied in this research is the material passed through a 19 mm (3/4 inch) mesh. Figure 2 provides a pavement material; the result of the soil granulation test performed according to the ASTM-D4226 standard. This soil is one of the materials used in pavement layers, especially as a base layer.

**Figure 3:**
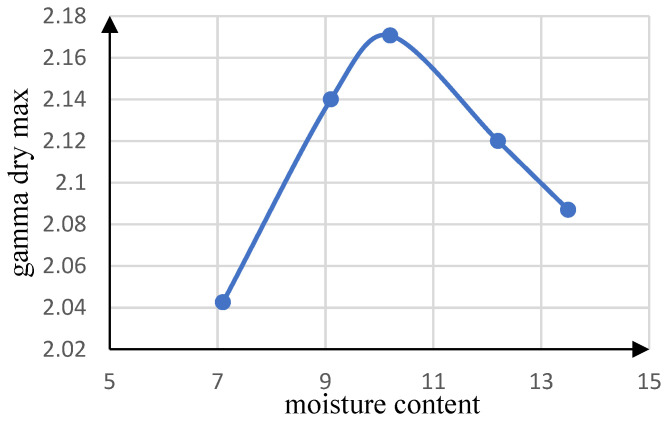
Standard compaction curve of the utilized soil.

Table 1 presents the engineering properties of this soil, which are approximately similar to the gradation characteristics of the base layer according to gradation D of the AASHTO M147 standard. To determine the optimal moisture percentage and the maximum dry density of the desired soil, the standard density test was performed for the used soil based on the ASTM-D698 standard. Figure 3 depicts the soil density diagram and presents the standard compaction curve of the utilized soil. As shown, the optimal moisture percentage is equal to 10% and the maximum dry density is equal to 2.17 g per cubic centimeter.

### 2.2. Zeolite

Zeolite is a mineral that is mainly composed of aluminosilicate. Regarding the special feature of zeolite in water absorption, this material has been widely used in various industries, including medicine, agriculture, the production of detergents, and construction. Zeolite is also used as a catalyst in the process of water purification. Therefore, civil projects are one of zeolite’s main and important applications [14,24].

Zeolite is a crystalline, hydrated aluminosilicate that consists of alkali and alkaline-earth metals. Zeolite is found abundantly in some natural resources of Iran and has pozzolanic properties. Considering the arrangement of atoms in zeolite, there are channels and cavities with fixed dimensions in the structure of the zeolite, which can be a storage place for water, gas, and other solid materials. Further, a wide range of cations such as $Na^{+}$, $K^{+}$, $Ca^{2+}$ and $Mg^{2+}$ can be placed in the cavities of zeolite, affecting the properties of zeolite.

**Figure 4:**
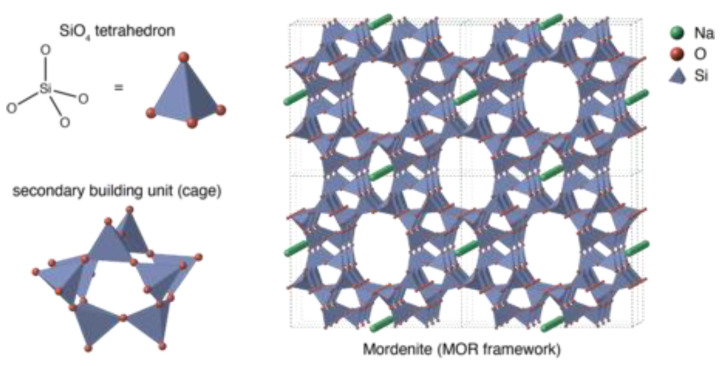
Chemical structure of zeolite [14,25].

Concerning the crystal shape, zeolites are divided into the following types: columnar, filamentous, and mixed crystals. From the geological point of view, natural zeolites are divided into two types: sedimentary and volcanic, and the ratio of silicon to aluminum is higher in sedimentary zeolites. The zeolites in Iran are mostly of sedimentary type. Figure 4 shows the microscopic structure of the zeolite. As observed, the pores in the zeolite structure are visible and their size is around 9 to 11 angstroms. Silicon-Oxygen Tetrahedron ($Si.O_{4}$), which is created by substituting one silicon atom in the center and four oxygen atoms around it, is the main base of the zeolite structure [14].

In recent years, zeolite has been widely used in various fields, due to its important characteristics such as high ion exchange capability, high water absorption capability, dehydration capability in the drying process, thermal stability, and high gas absorption capacity [15]. There are various types of zeolite, including Analcime, chabazite, clinoptilolite, hollandite, phillipsite, erionite, ferrierite, laumontite, and mordenite [26,27,28]. Natural zeolites are usually not pure and are mixed with other minerals, metals, quartz, or other zeolites.

The zeolite used in this research was of clinoptilolite type and was purchased from Asia Mines and Minerals Development Company. The zeolite, which was extracted from a mine located at a distance of 21 km from the north of Semnan city (Iran), was used in a size smaller than sieve NO. 200. Table 2 reports the physical and chemical properties of the zeolite. Moreover, the chemical analysis of zeolite is presented in Table 3. According to the location of the zeolite mine as well as the conditions and specifications of the processing of raw materials and the final product, mining companies try to produce a uniform product in terms of mechanical specifications. In other words, it can be expected that the product of a specific mine has fixed mechanical characteristics. In fact, it is rare that mineral materials are used without any processing in the manufacturing process.

### 2.3. Cement

So far, cement has been used as a common and basic stabilizer to improve the resistance performance of soil. The cement used in this research was Portland type II and was prepared by Sepahan Cement Company in Isfahan. Further, cement was produced according to ASTM C150, ISIRI 389, and EN 197-1 standards (equivalent to CEM I 42.5 N) in Sepahan cement company, whose physical and chemical characteristics are presented in Table 4 and Table 5, respectively.

### 2.4. Research Methods

To make a mixture of samples, we first mixed soil, cement, and zeolite with the desired weight ratios for each sample (according to Table 6), and then added water up to the optimal moisture percentage of the soil (equal to 10% of dry soil weight). After mixing water and materials, the mixtures were poured into cubic molds with dimensions of 10 × 10 × 10 cm and compacted to reach the specific weight equivalent to the optimal moisture percentage (according to the compaction test results). After treating the samples inside the mold for a few days, the samples were removed and the rest of their treatment period was completed. In this research, the values of zeolite replacement percentage (instead of cement) compared to the cement weight were equal to 0, 20, 30, 40, and 60%. These values were selected because the literature shows that more than 60 percent of zeolite instead of cement cannot be useful. Moreover, it is expected that the optimum zeolite replacement percentages instead of cement be in the 20 to 40% percentage. Moreover, the interval of 10 percent is a common range to change additives for such soil improvement options. Table 6 reports the characteristics of the samples made in this study based on the replacement percentages and treatment time. The percentage of adhesive materials (sum of the cement and zeolite) used in this research is considered to be a constant amount of 5%.

The samples were treated in 7- and 28-day intervals. For this purpose, a closed chamber with a temperature of 25 °C was used to provide the treatment conditions of the samples. Water was sprinkled on the surface of the samples at a constant rate during the treatment period, in order to keep the humidity of the samples constant. The unconfined compressive strength (UCS) test is one of the common and accepted tests for measuring the strength of stabilized soil samples, and it shows well the effect of stabilizing materials on the strength of mixed soil. This test is carried out according to the ASTM D2166-06 standard in the form of control stress and the values of force and displacement of the sample are recorded until the moment of sample failure. Regarding the recorded forces and displacements, the stress and strain values are then calculated and the stress versus strain diagram is drawn for the samples. The accuracy of the employed strain gauge is 0.01 mm and the accuracy of the employed load cell gauge is 0.01 kN. As the test apparatuses are implemented in a commercial laboratory, they have to be calibrated because that is important for the QC office of the laboratory and the clients to achieve reliable results.

As part of a common test plan in the soil mechanics laboratory, each test has 3 replicates. In other words, to determine the unconfined compressive strength of each sample at the desired states, 3 replicates were tested and the average value of the results was presented as the final value of that test.

The average values of replicates are often used to develop statistical models, and the resistance value of each replicate is not entered independently in statistical modeling. Therefore, the results are considered valid in terms of laboratory output, and the minimum requirements for the development of the mathematical model have also been included. As the test apparatuses are implemented in a commercial laboratory, they have to be calibrated because that is important for the QC office of the laboratory and the clients to achieve reliable results.

## 3. Results and Discussion

### 3.1. Unconfined Uniaxial Compressive Strength (UCS) and Failure Strain

**Figure 5:**
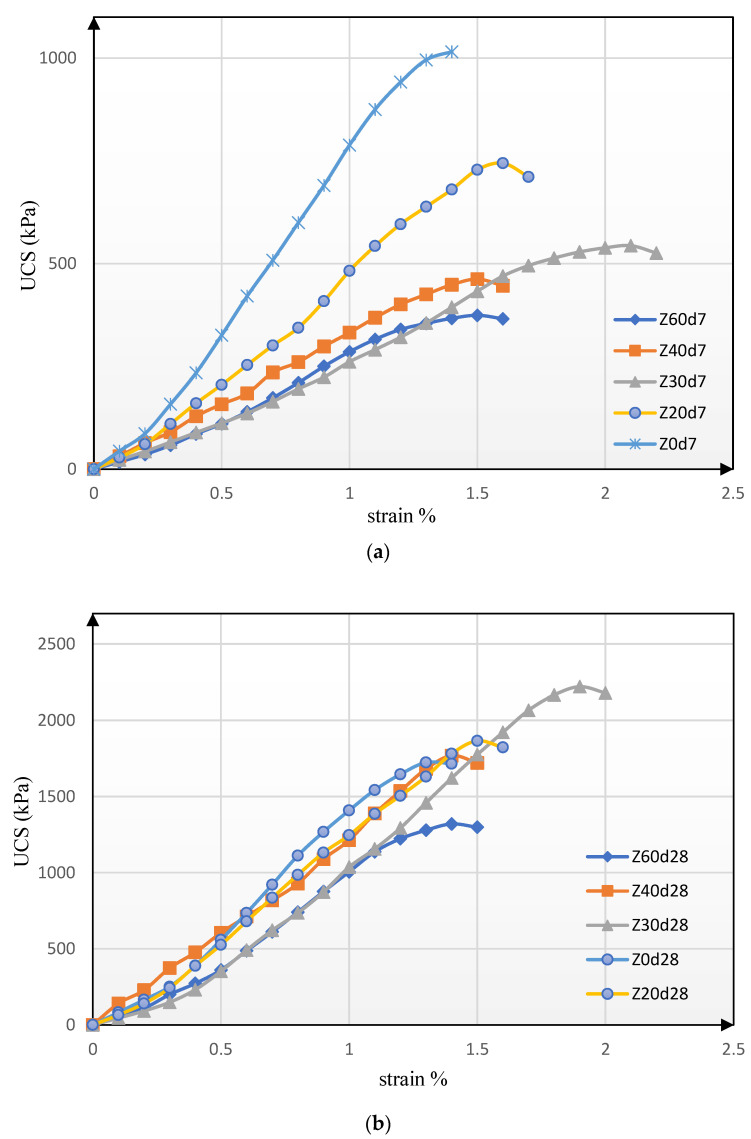
Stress-strain diagrams for different samples during a treatment period of (**a**) 7 days and (**b**) 28 days.

In the previous section, we mentioned that the percentage of stabilizing materials for all samples is equal to 5 wt.% of dry aggregate materials and thus, we added zeolite with amounts equal to 0, 20, 30, 40, and 60 wt.% of cement to different samples. The suggested amounts were according to the review of previous studies, and we omitted the use of larger amounts of zeolite, due to the decrease in strength in samples containing more than 60% zeolite. Figure 5 illustrates the graphs of changes in stress versus strain for samples containing different replacement percentages according to the unconfined compression test for treatment periods of 7 and 28 days.

As shown in Figure 5a, the sample without zeolite is ruptured at a stress of about 1000 kPa and a strain of about 1.4%. Accordingly, the sample has the lowest strain and the highest compressive strength. Concerning the samples with 20, 30, 40, and 60 wt.% of the cement, the ultimate unconfined compressive strength decreases by increasing percentages, although the conditions are slightly different in terms of strain variation. According to the curve related to the base layer soil sample containing 30% of zeolite (Z30d7), this sample showed a completely more flexible behavior and obtained the highest strain with a value of about 2.2% among all the 7-day samples. This issue indicates that the addition of zeolite could not increase the unconfined compressive strength in the short term, although it could affect the elastic behavior and produce a softer failure. Since the power of the pozzolanic reaction of zeolite is much lower than that of cement, the expectation of low speed to cause setting and adhesion for it will also be less. Cement has much higher reactivity than zeolite or even lime due to its much higher Blaine, as well as the heating in its production process. Therefore, it was not expected that zeolite could significantly increase the strength of the modified soil mixture in a short time; the results of the research tests also confirmed this.

On the other hand, changes in stress-strain curves in Figure 5b have different conditions compared to Figure 5a. As observed in Figure 5b, increasing the treatment period and consequently, pozzolanic reactions, approaches the stress-strain variation rates so that almost all the samples have a slope of the line close to each other, despite the lack of uniform proportion in the placement order of the curves with the amount of replacement percentage (purely ascending or purely descending). In other words, increasing the percentage of zeolite instead of cement from 0 to 30% increases unconfined compressive strength, although a further increase in the replacement rate of zeolite to 60% of the cement weight decreases the unconfined compressive strength. This issue indicates that a 30% replacement of zeolite instead of cement causes the greatest increase in compressive strength. On the other hand, reviewing strain changes indicates that the base soil containing a 30% replacement of cement with zeolite has a significantly more flexible behavior, and the sample containing 60% zeolite gains the highest strain, unlike other samples that have a strain of 1.5 to 1.6%.

**Figure 6:**
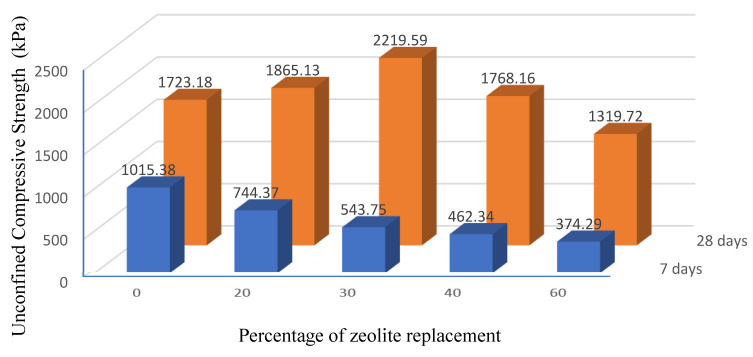
UCS values as a function of the zeolite replacement percentage.

**Figure 7:**
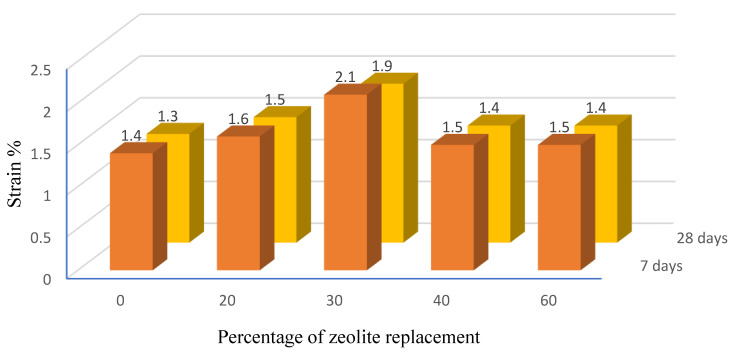
Values of ultimate strain against zeolite replacement percentage instead of cement.

Figure 6 and Figure 7 show and compare the variation trends of unconfined uniaxial compressive strength and strain in the samples for different percentages of using zeolite instead of cement for 7 and 28 days, respectively.

**Figure 8:**
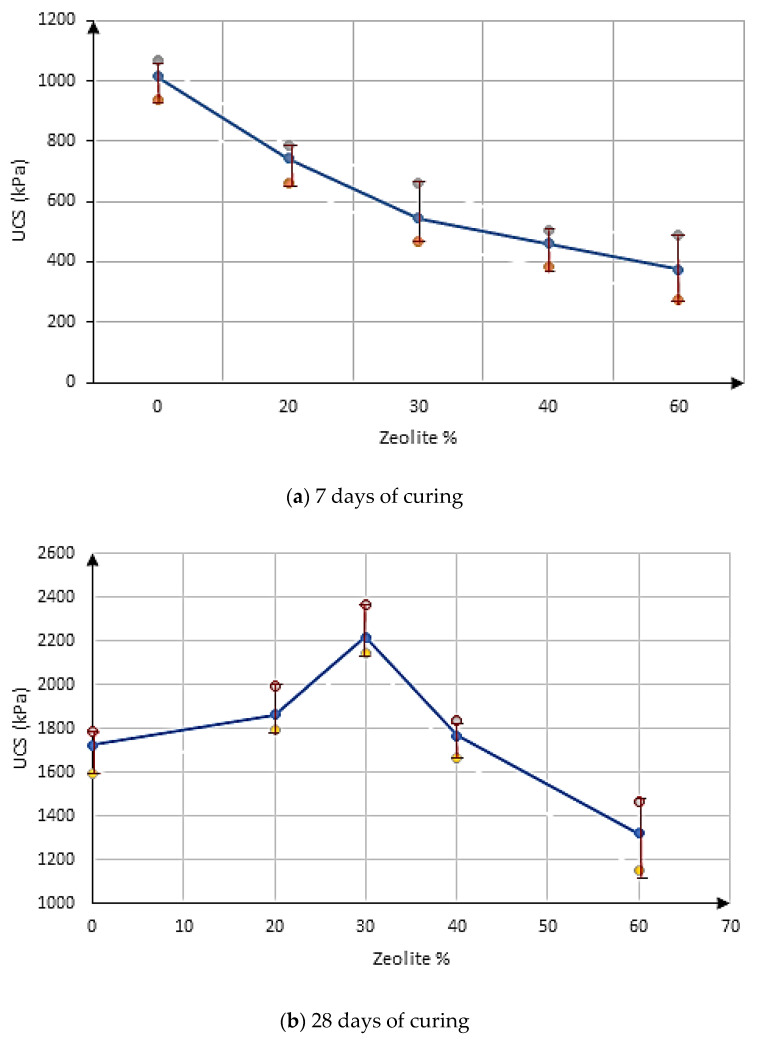
The range bars for changes of USC in different percentages of zeolite as a replacement to cement: (**a**) 7 days, (**b**) 28 days.

Figure 8 represents the range bars of replicates’ UCS and the variation trends of UCS in the samples for different percentages of using zeolite instead of cement for 7 and 28 days of curing, respectively.

By quantitatively analyzing the results of Figure 6, it is found that the samples containing zeolite have lower compressive strength after 7 days’ treatment compared to the sample without zeolite. In other words, increasing the percentage of zeolite replacing the cement decreases compressive strength in the samples treated for seven days. Based on the results, an increase in the replacement percentage of zeolite from 0 to 30 wt.% of cement decreases the compressive strength by 46%, while a further increase in the replacement percentage of zeolite from 30 to 60% decreases the compressive strength by about 30%. This reduction in strength shows that the hydration reactions in zeolite, as an alternative pozzolanic material, could not be performed well and quickly within 7 days’ treatment. As zeolite is a raw natural material implemented with the fewest production procedures, its hydration power is low and it may take a long duration to react sufficiently. This means that on the one hand, zeolite is not expected to improve the resistance of the modified soil mixture in a short time. On the other hand, since the size of used zeolite is smaller than sieve No. 200, it is virtually unable to tolerate or transmit tensions; in other words, it just has the role of filler material and requires more time to activate hydration and obtain adhesion.

The results of the samples treated for 28 days in Figure 6 highlight that increasing the replacement percentage of zeolite instead of cement to up to 30% of the cement weight increases the compressive strength so that the highest compressive strength after 28 days’ treatment is obtained in the sample containing 30% of zeolite. The unconfined compressive strength in this sample is increased by 28.8% compared to the sample without zeolite (containing only 5% cement). However, a further increase in the replacement percentage of zeolite instead of cement leads to a downward trend in compressive strength.

It should be noted that adding cement to the soil increases the hydration activity and reduces the moisture in the soil and cement mixture. Therefore, the addition of cement to the soil causes brittle fracture strains in the soil, which is one of the problems of soil stabilization with cement. Figure 7 displays the failure strain values for different samples. According to the results, the highest failure strain is related to samples containing 30% zeolite as a replacement to cement. Thus, the sample containing 30% zeolite will have a softer failure than that of the sample with only cement.

### 3.2. SEM-EDX Analysis

**Figure 9:**
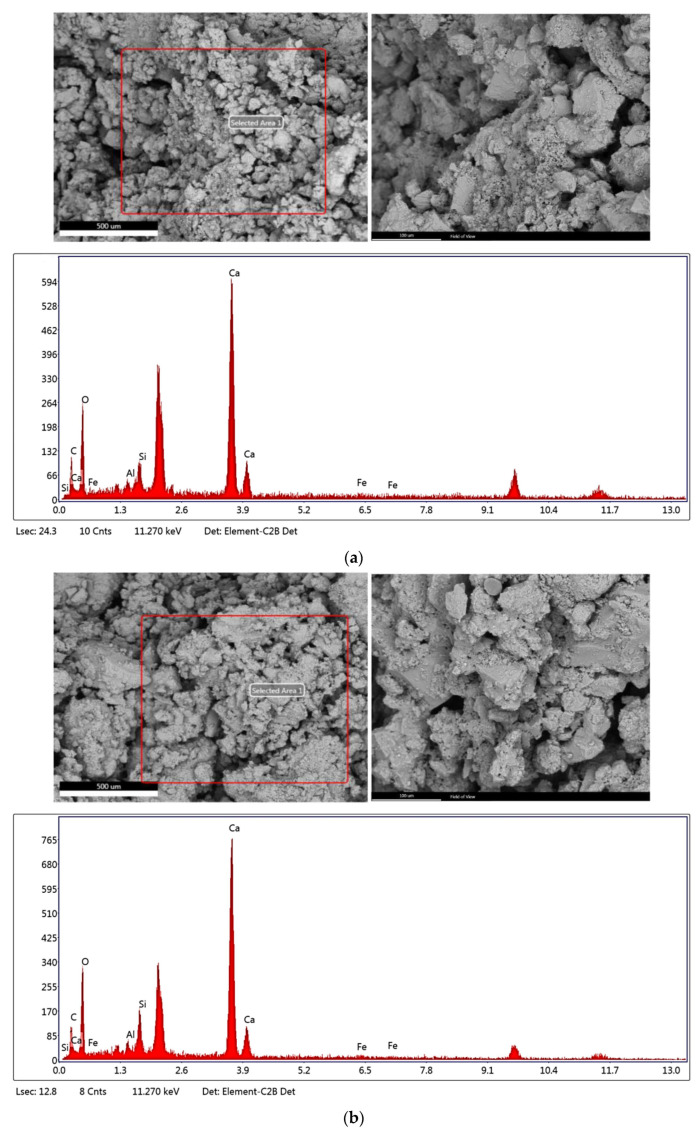

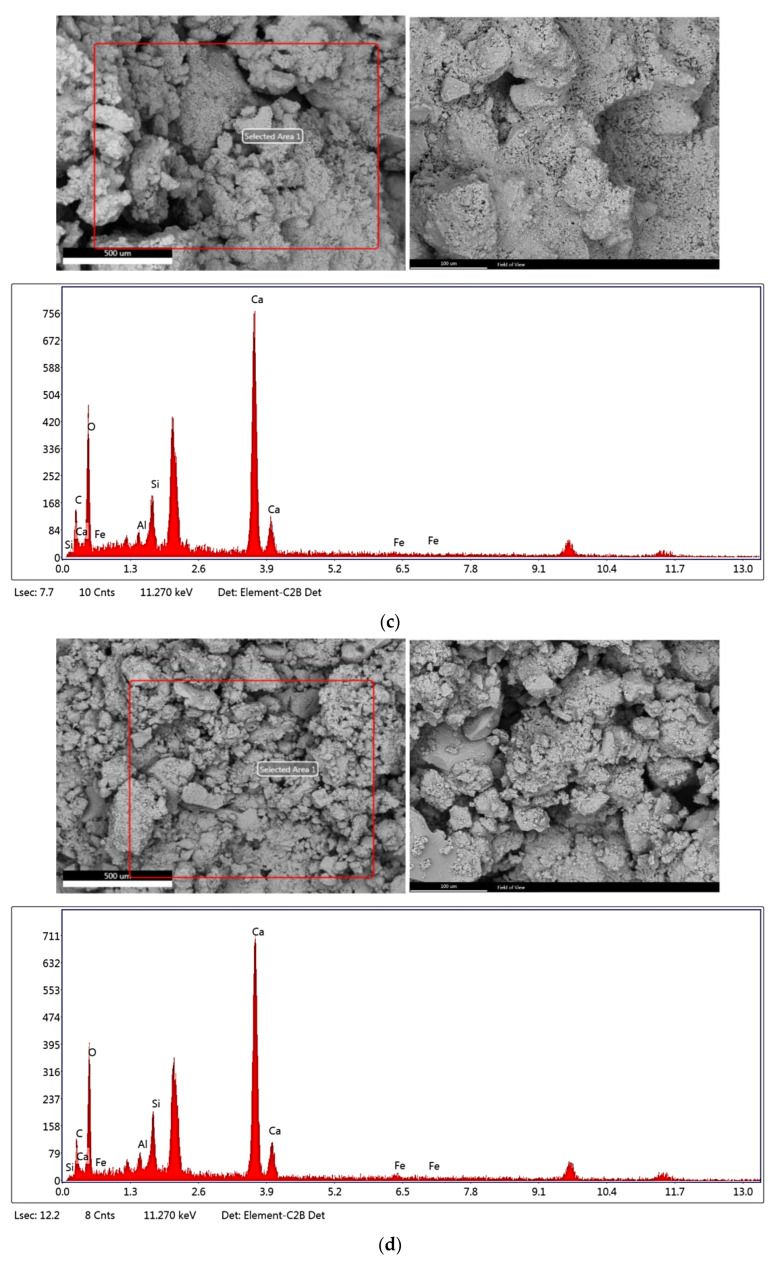
SEM images and EDAX analysis for different percentages of zeolite replacing cement and with 28 days’ treatment. (**a**) Zero replacement of zeolite instead of cement, (**b**) 20% replacement of zeolite instead of cement, (**c**) 30% replacement of zeolite instead of cement, (**d**) 40% replacement of zeolite instead of cement.

In this study, scanning electron microscopy (SEM) was used to investigate the morphological structure of cement-stabilized soil before and after replacing with different percentages of zeolite. SEM analysis was performed to observe the conditions of adding zeolite to the soil instead of cement on a microscopic scale. Figure 9 provides the results of scanning samples containing 0, 20, 30, and 40% replacement of zeolite instead of cement for 28 days‘ treatment with a drawing scale of 500 and 100 μm. These pictures clearly show the soil grains. As observed, most of the soil grains have broken surfaces and the black spots in the image represent the holes. Moreover, parts in the form of frost on the surface of the soil grains show the calcites caused by the hydration of pozzolanic materials, including zeolite and cement in the sample, which was created after the treatment period. Generally, the higher the number of binds created due to pozzolanic activity (which forms a more continuous network), the higher the compressive strength of the samples will be. As shown, the sample containing zeolite (30% replacement of the cement weight with zeolite) has the highest amount of the created calcite compared to other samples. In this sample, calcites completely cover the surface of the soil grains. In addition, the volume of cavities observed in this sample is the lowest compared to other samples, and consequently, it is quite reasonable to reach the maximum compressive strength. The results are in good agreement with recent research [19].

Further, the elemental analysis of the samples was performed using the energy-dispersive X-ray (EDX) method to investigate the effect of pozzolanic materials on the strength of the samples. As observed, oxygen and calcium are the most abundant in the samples, with amounts of aluminum, silicon, iron, and carbon.

### 3.3. X-ray Diffraction (XRD)

**Figure 10:**
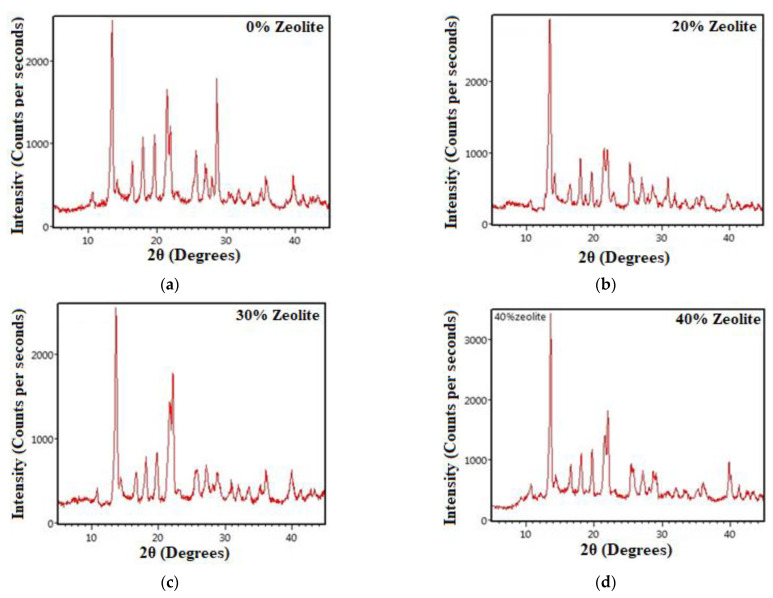
XRD analysis spectrum for samples containing different replacement percentages of zeolite to cement (**a**) 0%, (**b**) 20%, (**c**) 30%, and (**d**) 40% (wt.% of cement).

To better understand the chemical compounds in the samples, the XRD test was performed for the samples containing 0 to 40% zeolite, the results of which are shown in Figure 10.

The results of the XRD test as a mineralogical analysis show that the single-phase structure of calcite matches well with the samples. The hexagonal CaCO3 calcite is the dominant phase in these samples. Given the sample containing 30% of replaced zeolite, the intensity of calcite formed with a hexagonal structure is higher, which indicates the good performance of pozzolanic reactions. The results of the XRD test are in good agreement with those of the EDAX test.

### 3.4. The Significance Level of Test Results

**Figure 11:**
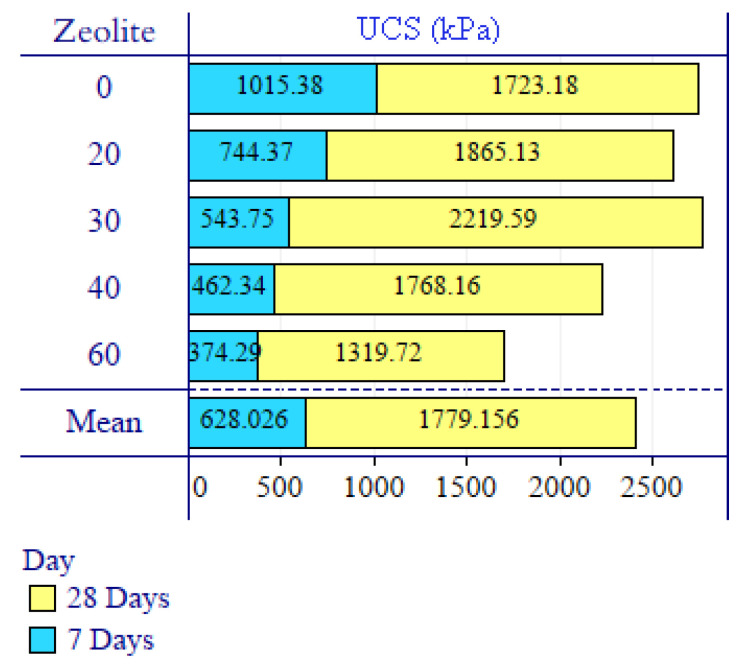
Data entered in statistical analysis.

In this research, the results were analyzed using statistical methods to determine the effectiveness of the variables of zeolite percentage and treatment time, as well as to evaluate the accuracy of the results. In line with the data analysis in the general linear model, the two-factor analysis of variance test was used to determine the effect of the mentioned variables on the UCS kPa variable. If the analysis of the variance test was significant, Bonferroni’s post hoc test was used to determine the differences between groups. Figure 11 presents the input data related to the unconfined uniaxial compressive strength of the samples treated at 7 and 28 days. Table 7 reports the results of the statistical analysis.

### 3.5. Developing a Mathematical Model for the Compressive Strength of Stabilized Soil

Regarding the time-consuming nature and costly process of these tests, it is crucial to develop a model that can estimate unconfined compressive strength values in other percentages of zeolite. In this regard, a model was fitted to the data using Eureqa software. The parameters of the model include the replacement percentage of zeolite to cement in a range of 0 to 5% of the weight of dry soil materials, along with the duration of treatment (time intervals of 1 to 28 days). The analysis performed on the model reveals that the model of Equation (2) can accurately estimate the value of unconfined compressive strength for different replacement percentages of zeolite to cement for various treatment periods. Table 8 gives the parameters of statistical analysis and the goodness of fit of the model.

(1) Z+C=5      0%<Z,C<5%

(2) $$\mathrm{UCS}=734.5+57.7*T+\;Z*T\;+14.13*T*\cos\left(235.994*Z\;+14.134{*T}^{2}\right)\;-220.613*Z\;-13.92*T\;-57.7*Z*\cos\left(T\right)$$

where C represents the cement percentage in terms of the weight of the dry material (0 to 5%), Z indicates the zeolite percentage in terms of the weight of the dry material (0 to 5%), T shows the treatment time (1 to 28 days), and UCS is the unconfined compressive strength in kilopascals.

**Figure 12:**
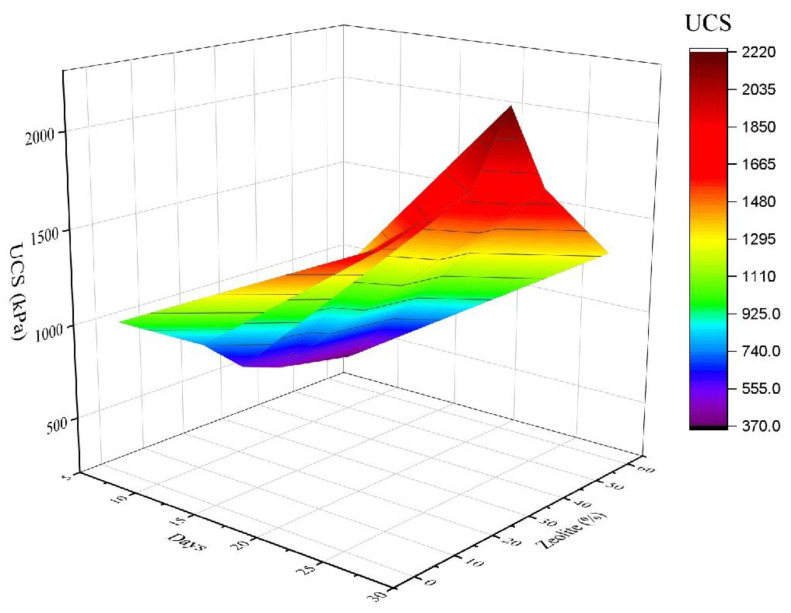
Continuous three-dimensional procedure of UCS values versus percentage of replaced zeolite and treatment times for laboratory results and values estimated from the model.

Figure 12 shows the three-dimensional diagram of the continuous procedure related to the UCS values versus the zeolite percentage and the treatment time for the values estimated from the model. As observed, there is a good agreement between the experimental results and the data estimated from the model. The accuracy of the model was verified using the supplementary tests for a random state of zeolite percentage and treatment model. To examine the prediction accuracy of the proposed regression model, some supplementary tests were implemented which are presented in Table 8. The given results in Table 8 satisfy that the proposed model statistically fits to the data. For example, the $R^{2}$ value is more than 99.59%, which means observed outcomes are replicated by the proposed regression model very well.

## 4. Conclusions

As mentioned, various efforts have been made to replace natural pozzolanic materials instead of cement. So far, cement has been highly used in stabilizing soils used in different layers of road construction such as subgrade or sub-base, which is considered one of the most important applications of this material. Zeolite is a mineral material with pozzolanic properties, which is mainly composed of aluminosilicate and has the potential to replace a part of the cement required in soil stabilization. This research sought to evaluate the effect of replacing 0 to 60% zeolite instead of cement used in soil stabilization, by conducting an unconfined compressive strength test. The results of the present research are explained in the following.

- For all soil samples on the pavement base layer containing different percentages of zeolite replacing the cement within 7 days of treatment, the unconfined compressive strength was lower than that of the sample without zeolite (or in other words, stabilized with only 5% cement). For a 7-day treatment period, replacing 30% cement with zeolite with 7 days’ treatment increases the failure strain by 50% compared to the sample without zeolite.
- The soil sample containing 30% zeolite replacing the cement with 28 days’ treatment obtained the maximum unconfined compressive strength among all the studied samples. A further increase in the replacement percentage beyond 30% decreases the trend of changes in compressive strength.
- After 28 days’ treatment, replacing 30% zeolite instead of cement increased the failure strain by 41% compared to the sample without zeolite and obtained the maximum failure strain among the samples containing other percentages of zeolite.
- Samples stabilized with a combination of zeolite and cement in some cases have higher unconfined compressive strength relative to samples stabilized with only cement or only zeolite.
- In general, the addition of zeolite softened the failure behavior of the samples and increased the failure strain compared to the samples without zeolite.
- Increasing the duration of the sample treatment period increases the compressive strength and slightly decreases the failure strain in the samples.
- In general, the addition of zeolite (except 60% replacement) to samples with a 28-day treatment period can increase the compressive strength compared to the sample without zeolite. Based on the results, the minimum and maximum increase in strength are equal to 2.6 and 28.8% for the samples containing 40 and 30% zeolite, compared to the sample without zeolite (samples containing only cement), respectively.

## Figures and Tables

**Table 1:** Characteristics of the employed soil as the base layer material.

Characteristic	Value	Test Standard No.	Requirements Range
Plasticity index	3%	AASHTO T 90	Maximum 4%
Liquid limit	23%	ASTM D4318	Maximum 25%
Sand equivalent	55%	AASHTO T 176	Minimum 40%
CBR	83%	AASHTO T 193	Minimum 80%

**Table 2:** Physical and chemical properties of the zeolite.

Characteristics	Value
Specific gravity ($\mathrm{gr}/{\mathrm{cm}}^{3}$)	1.19
Specific surface area ($\mathrm{gr}/{\mathrm{cm}}^{3}$)	1000
Specific gravity of aggregate ($G_{s}$)	2.2
Ion exchange capacity (gr/meq)	2.6

**Table 3:** Chemical analysis of zeolite.

L.O.I	NaCl	SrO	$TiO_{2}$	$Al_{2}O_{3}$	$Fe_{2}O_{3}$	CaO	MgO	$Na_{2}O$	$SO_{3}$	$SiO_{2}$
8.3%	1.3%	0.1%	0.3%	10.9%	1.8%	0.5%	0.6%	2.7%	0.4%	73.1%

**Table 4:** Physical properties of Portland cement (Type II).

Physical Properties	Blaine $\left(cm^{2}/gr\right)$	Initial Setting (min)	Final Setting (min)	Compressive Resistance $\left(kg/cm^{2}\right)$				Autoclave Expansion (%)
				2 Days	3 Days	7 Days	28 Days	
Sepahan cement	3500	150	210	180	250	410	550	0.08
Standard Deviation	100	30	30	15	20	25	25	0.03
ISIRI	>2800	>45	<360	-	>100	>175	>315	<0.8
EN	-	>60	-	>100	-	-	>425	-

**Table 5:** Chemical properties of Portland cement (Type II).

Chemical Parameters	$SiO_{2}\;(\%)$	$Al_{2}O_{3}\;(\%)$	$Fe_{2}O_{3}\;(\%)$	CaO (%)	MgO (%)	Cl (%)	$SO_{3}\;(\%)$	L.O.I (%)	I.R (%)	Free CaO (%)	Total Alkalis (%)	$C_{3}A\;(\%)$	$Cr^{+6}\;(\%)$
Sepahan cement	21.1	4.9	4	64.2	2.2	0.025	2.2	1	0.35	1.3	0.75	6	<0.009
Standard Deviation	0.5	0.2	0.2	0.7	0.2	0.005	0.2	0.3	0.2	0.3	0.05	1	-
ISIRI	>20	<6	<6	-	<5	-	<3	<3	<0.75	-	-	<8	-
EN	-	-	-	-	-	<0.1	<3.5	<5	<5	-	-	-	-

**Table 6:** Naming, mixing ratios, and treatment time of the tested samples.

Sample No.	Sample Code	Percentage of Stabilizing Materials Relative to the Dry Soil Weight		Treatment Period
		Cement (%)	Zeolite (%)	
1	Z0d7	5	0	7 Days
2	Z20d7	4	1	7 Days
3	Z30d7	3.5	1.5	7 Days
4	Z40d7	3	2	7 Days
5	Z60d7	2	3	7 Days
6	Z0d28	5	0	28 Days
7	Z20d28	4	1	28 Days
8	Z30d28	3.5	1.5	28 Days
9	Z40d28	3	2	28 Days
10	Z60d28	2	3	28 Days

**Table 7:** The results of the two-factor analysis of variance for zeolite and day variables.

Source	Type III Sum of Squares	df	Mean Square	F	Sig.	Partial Eta Squared
Corrected Model	3,721,462.344 ^a^	5	744,292.469	11.046	0.019	0.932
Intercept	14,486,312.950	1	14,486,312.950	215.000	0.000	0.982
Days	3,312,750.692	1	3,312,750.692	49.166	0.002	0.925
Zeolite	408,711.652	4	102,177.913	1.516	0.348	0.603
Error	269,512.948	4	67,378.237			
Total	18,477,288.240	10				
Corrected Total	3,990,975.292	9				

^a^. R Squared = 0.932 (Adjusted R Squared = 0.848).

**Table 8:** Statistical analysis of the model fitted to the unconfined compressive strength data.

Parameter	Value
$R^{2}$ Goodness of Fit	0. 99596159
Correlation Coefficient	0.99800283
Maximum Error	160.48331
Mean Squared Error	2265.1063
Mean Absolute Error	29.713533
Coefficients	8
Complexity	53
Primary Objective	29.713533
Fit (Normalized Primary Obj.)	0.044481804

## Data Availability

Access to data is restricted, except for the revealed data in the manuscript. Some or all data are available from the corresponding author upon request.
